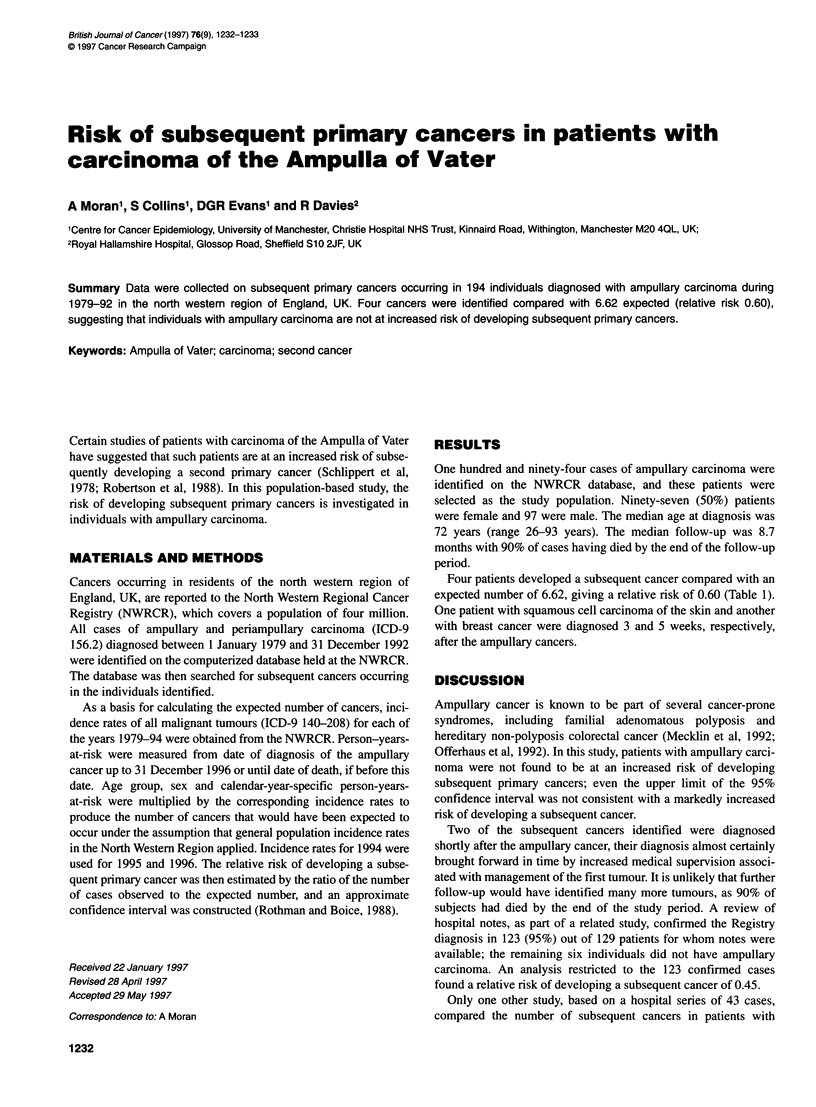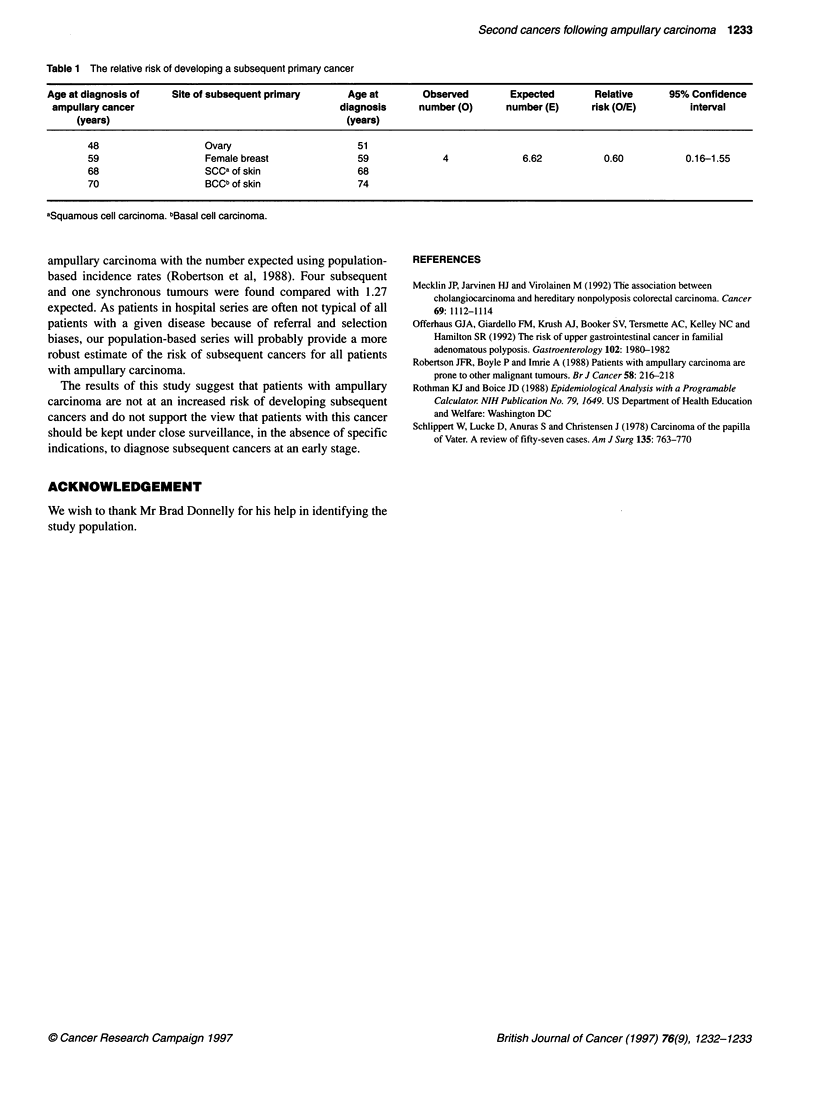# Risk of subsequent primary cancers in patients with carcinoma of the Ampulla of Vater.

**DOI:** 10.1038/bjc.1997.539

**Published:** 1997

**Authors:** A. Moran, S. Collins, D. G. Evans, R. Davies

**Affiliations:** Centre for Cancer Epidemiology, University of Manchester, Christie Hospital NHS Trust, UK.

## Abstract

Data were collected on subsequent primary cancers occurring in 194 individuals diagnosed with ampullary carcinoma during 1979-92 in the north western region of England, UK. Four cancers were identified compared with 6.62 expected (relative risk 0.60), suggesting that individuals with ampullary carcinoma are not at increased risk of developing subsequent primary cancers.


British Journal of Cancer (1997) 76(9), 1232–1233
© 1997 Cancer Research Campaign

### Risk of subsequent primary cancers in patients with carcinoma of the Ampulla of Vater

**A Moran[1], S Collins[1], DGR Evans[1] and R Davies[2]**

[1]Centre for Cancer Epidemiology, University of Manchester, Christie Hospital NHS Trust, Kinnaird Road, Withington, Manchester M20 4QL, UK;
[2]Royal Hallamshire Hospital, Glossop Road, Sheffield S10 2JF, UK

**Summary** Data were collected on subsequent primary cancers occurring in 194 individuals diagnosed with ampullary carcinoma during 1979–92 in the north western region of England, UK. Four cancers were identified compared with 6.62 expected (relative risk 0.60), suggesting that individuals with ampullary carcinoma are not at increased risk of developing subsequent primary cancers.

**Keywords:** Ampulla of Vater; carcinoma; second cancer

Certain studies of patients with carcinoma of the Ampulla of Vater have suggested that such patients are at an increased risk of subsequently developing a second primary cancer (Schlippert et al, 1978; Robertson et al, 1988). In this population-based study, the risk of developing subsequent primary cancers is investigated in individuals with ampullary carcinoma.

### MATERIALS AND METHODS

Cancers occurring in residents of the north western region of England, UK, are reported to the North Western Regional Cancer Registry (NWRCR), which covers a population of four million. All cases of ampullary and periampullary carcinoma (ICD-9 156.2) diagnosed between 1 January 1979 and 31 December 1992 were identified on the computerized database held at the NWRCR. The database was then searched for subsequent cancers occurring in the individuals identified.

As a basis for calculating the expected number of cancers, incidence rates of all malignant tumours (ICD-9 140–208) for each of the years 1979–94 were obtained from the NWRCR. Person–years-at-risk were measured from date of diagnosis of the ampullary cancer up to 31 December 1996 or until date of death, if before this date. Age group, sex and calendar-year-specific person-years-at-risk were multiplied by the corresponding incidence rates to produce the number of cancers that would have been expected to occur under the assumption that general population incidence rates in the North Western Region applied. Incidence rates for 1994 were used for 1995 and 1996. The relative risk of developing a subsequent primary cancer was then estimated by the ratio of the number of cases observed to the expected number, and an approximate confidence interval was constructed (Rothman and Boice, 1988).

*Received 22 January 1997*
*Revised 28 April 1997*
*Accepted 29 May 1997*

*Correspondence to:* A Moran

### RESULTS

One hundred and ninety-four cases of ampullary carcinoma were identified on the NWRCR database, and these patients were selected as the study population. Ninety-seven (50%) patients were female and 97 were male. The median age at diagnosis was 72 years (range 26–93 years). The median follow-up was 8.7 months with 90% of cases having died by the end of the follow-up period.

Four patients developed a subsequent cancer compared with an expected number of 6.62, giving a relative risk of 0.60 (Table 1). One patient with squamous cell carcinoma of the skin and another with breast cancer were diagnosed 3 and 5 weeks, respectively, after the ampullary cancers.

### DISCUSSION

Ampullary cancer is known to be part of several cancer-prone syndromes, including familial adenomatous polyposis and hereditary non-polyposis colorectal cancer (Mecklin et al, 1992; Offerhaus et al, 1992). In this study, patients with ampullary carcinoma were not found to be at an increased risk of developing subsequent primary cancers; even the upper limit of the 95% confidence interval was not consistent with a markedly increased risk of developing a subsequent cancer.

Two of the subsequent cancers identified were diagnosed shortly after the ampullary cancer, their diagnosis almost certainly brought forward in time by increased medical supervision associated with management of the first tumour. It is unlikely that further follow-up would have identified many more tumours, as 90% of subjects had died by the end of the study period. A review of hospital notes, as part of a related study, confirmed the Registry diagnosis in 123 (95%) out of 129 patients for whom notes were available; the remaining six individuals did not have ampullary carcinoma. An analysis restricted to the 123 confirmed cases found a relative risk of developing a subsequent cancer of 0.45.

Only one other study, based on a hospital series of 43 cases, compared the number of subsequent cancers in patients with

**Table 1** The relative risk of developing a subsequent primary cancer

| Age at diagnosis of ampullary cancer (years) | Site of subsequent primary | Age at diagnosis (years) | Observed number (O) | Expected number (E) | Relative risk (O/E) | 95% Confidence interval |
|---|---|---|---|---|---|---|
| 48 | Ovary | 51 | | | | |
| 59 | Female breast | 59 | 4 | 6.62 | 0.60 | 0.16–1.55 |
| 68 | SCC[a] of skin | 68 | | | | |
| 70 | BCC[b] of skin | 74 | | | | |

[a]Squamous cell carcinoma. [b]Basal cell carcinoma.

ampullary carcinoma with the number expected using population-based incidence rates (Robertson et al, 1988). Four subsequent and one synchronous tumours were found compared with 1.27 expected. As patients in hospital series are often not typical of all patients with a given disease because of referral and selection biases, our population-based series will probably provide a more robust estimate of the risk of subsequent cancers for all patients with ampullary carcinoma.

The results of this study suggest that patients with ampullary carcinoma are not at an increased risk of developing subsequent cancers and do not support the view that patients with this cancer should be kept under close surveillance, in the absence of specific indications, to diagnose subsequent cancers at an early stage.

### ACKNOWLEDGEMENT

We wish to thank Mr Brad Donnelly for his help in identifying the study population.

### REFERENCES

Mecklin JP, Jarvinen HJ and Virolainen M (1992) The association between cholangiocarcinoma and hereditary nonpolyposis colorectal carcinoma. *Cancer* **69**: 1112–1114

Offerhaus GJA, Giardello FM, Krush AJ, Booker SV, Tersmette AC, Kelley NC and Hamilton SR (1992) The risk of upper gastrointestinal cancer in familial adenomatous polyposis. *Gastroenterology* **102**: 1980–1982

Robertson JFR, Boyle P and Imrie A (1988) Patients with ampullary carcinoma are prone to other malignant tumours. *Br J Cancer* **58**: 216–218

Rothman KJ and Boice JD (1988) *Epidemiological Analysis with a Programable Calculator. NIH Publication No. 79, 1649.* US Department of Health Education and Welfare: Washington DC

Schlippert W, Lucke D, Anuras S and Christensen J (1978) Carcinoma of the papilla of Vater. A review of fifty-seven cases. *Am J Surg* **135**: 763–770

© Cancer Research Campaign 1997